# Alcohol Use Disorder and Antisocial and Borderline Personality Disorders

**DOI:** 10.35946/arcr.v40.1.05

**Published:** 2019-12-30

**Authors:** Ashley C. Helle, Ashley L. Watts, Timothy J. Trull, Kenneth J. Sher

**Affiliations:** Ashley C. Helle, Ph.D., is a Postdoctoral fellow in the Department of Psychological Sciences, University of Missouri-Columbia, Columbia, Missouri; Ashley L. Watts, Ph.D., is a Postdoctoral fellow in the Department of Psychological Sciences, University of Missouri-Columbia, Columbia, Missouri.; Timothy J. Trull, Ph.D., is a Curators’ Distinguished Professor and a Byler Distinguished Professor in the Department of Psychological Sciences, University of Missouri-Columbia, Columbia, Missouri; Kenneth J. Sher, Ph.D., is a Curators’ Distinguished Professor in the Department of Psychological Sciences, University of Missouri-Columbia, Columbia, Missouri

**Keywords:** alcohol use disorder, antisocial personality disorder, borderline personality disorder, comorbidity

## Abstract

Alcohol use disorder (AUD) frequently co-occurs with other psychiatric disorders, including personality disorders, which are pervasive, persistent, and impairing. Personality disorders are associated with myriad serious outcomes, have a high degree of co-occurrence with substance use disorders, including AUD, and incur significant health care costs. This literature review focuses on co-occurring AUD and personality disorders characterized by impulsivity and affective dysregulation, specifically antisocial personality disorders and borderline personality disorders. Prevalence rates, potential explanations and causal models of co-occurrence, prognoses, and the status of existing treatment research are summarized. Several important future research considerations are relevant to these complex, co-occurring conditions. Research assessing mechanisms responsible for co-occurring AUD and antisocial personality disorder or borderline personality disorder will further delineate the underlying developmental processes and improve understanding of onset and courses. In addition, increased focus on the efficacy and effectiveness of treatments targeting underlying traits or common factors in these disorders will inform future prevention and treatment efforts, as interventions targeting these co-occurring conditions have relatively little empirical support.

## Introduction

The quest to understand the etiology, course, and treatment of alcohol use disorder (AUD) has given rise to an extensive body of work on identifying factors that contribute to these phenomena. Many of these factors, such as temperament and personality traits, are common to multiple psychiatric conditions, and some, such as variants of alcohol metabolizing genes, are specific to AUD. This review describes the co-occurrence of AUD with antisocial personality disorder (ASPD) and borderline personality disorder (BPD). The prevalence and effects of these personality disorders, their co-occurrence with AUD through the lens of several current models, and the treatment and overall implications of these complex co-occurrences are discussed.

The conceptualization and diagnostic criteria for AUD has evolved over the years and through editions of the *Diagnostic and Statistical Manual of Mental Disorders* (DSM). For example, in the text revision of the fourth edition of the DSM (DSM-IV-TR) the conceptualization included alcohol abuse and dependence, which were categories that comprised two different symptom sets and required a number of criteria for diagnosis.^1^ More recent conceptualizations of AUD are seen in the fifth edition of the DSM (DSM-5), which describes AUD as a single disorder with 11 criteria and includes a severity gradient designated by the number of criteria met (e.g., two to three symptoms constitute mild AUD).^2^ Although this conceptualization inherently is still categorical, the changes are consistent with a transition toward dimensional approaches (e.g., severity can be graded across one set of symptoms).^3^ Additional work needs to be done to capture a fully dimensional diagnosis for AUD.

Other diagnostic systems, such as the 11th revision of the *International Classification of Diseases* (ICD-11), have implemented new conceptualizations of AUD that differ from the alcohol abuse and dependence categories and that attempt to capture potential features of severity (e.g., harmful use diagnosis and recurrent problems).^4^ Note that many of the studies reported in this review focus on previous DSM conceptualizations of AUD, such as the categories of alcohol abuse and dependence from the DSM-IV-TR. In addition, much of the work described here conceptualizes AUD as a categorical diagnosis, either present or absent, although support for a categorical AUD taxonomy is declining.^1^ Differing AUD conceptualizations may affect the general consensus of research findings.

Personality disorder diagnoses and, more generally, psychopathology are migrating toward a dimensional classification system. For example, the ICD-11 includes a dimensional approach to personality disorder diagnosis.^4^ For classifying personality disorders, there has been a call for and transition to dimensional approaches, and a number of the proposed models largely align with robust and well-validated models of personality.^5^–^8^ The DSM-IV-TR personality disorder categories were retained in the DSM-5, but the DSM-5 (Section III: Emerging Measures and Models) proposes a new model that integrates dimensional aspects (e.g., dimensional personality traits) into a more traditional categorical classification model.^2^ This hybrid categorical-dimensional model, the alternative DSM-5 model for personality disorders, is described in more detail in the following section.

## Personality Disorders

Although the long-standing research aimed at identifying an “alcoholic personality”^9^ has not been particularly fruitful, these efforts have nevertheless identified some personality traits, or constellations thereof, that are associated with increased risk for alcohol use and misuse. ASPD and BPD, both characterized by impulsivity, negative emotionality, and antagonism, are two such constellations. This review focuses on ASPD and BPD; however, personality disorders in general are the focus of some research presented and are noted throughout.

ASPD is characterized by behavior patterns that show a lack of regard for and violation of the rights of others, deceit, manipulation, and impulsivity that have occurred since age 15, in addition to evidence of conduct disorder before age 15.^2^ BPD is conceptualized as a disorder of emotion dysregulation, impulsivity, suicidality, identity disturbance, and difficulties in interpersonal relationships. Although the DSM-5 classifies personality disorders categorically, the DSM-5 alternative, hybrid dimensional-categorical model of personality disorder describes these disorders in terms of broad personality domains (negative affectivity, detachment, antagonism, disinhibition, and psychoticism) and facets that are largely consistent with popular models of general personality, namely the five-factor model (see the section Trait Explanations for a detailed explanation of this model).^5^ Individual personality disorders such as BPD are then characterized by specific traits, resulting in a hybrid model that describes the disorders in terms of both dimensional trait features (e.g., disinhibition) and categories (e.g., BPD).

Within the alternative DSM-5 model for personality disorders, ASPD and BPD are characterized by high levels of disinhibition, with BPD additionally associated with high levels of negative affectivity, and ASPD additionally associated with high levels of antagonism. The ICD-11 conceptualizes personality disorders in a manner similar to the DSM-5 alternative model, such that dimensional traits (e.g., negative affectivity and disinhibition) are included in the diagnosis.^4^ Further, in the ICD-11, these traits accompany a general diagnosis for mild, moderate, or severe personality disorder.

### Prevalence

Epidemiological, community, and clinical psychiatric samples across all 10 categorical personality disorders have yielded prevalences ranging from 9% to 21% in community (nonclinical) samples^10^ to approximately 31% in psychiatric outpatient samples,^11^ with many individuals receiving diagnoses of more than one personality disorder. Across epidemiological studies, community prevalences for ASPD and BPD, individually, range from 1% to 4% and 1% to 6%, respectively.^10^

ASPD and BPD manifest in a broad array of maladaptive behaviors, including suicide, self-harm, aggression, criminal behavior, and substance misuse. Moreover, ASPD and BPD are associated with profound economic costs.^12^–^15^ ASPD is associated with criminal offenses, with ASPD prevalence as large as 60% in prison populations,^12^ and BPD is associated with higher suicide rates than those among the general population.^13^ Both conditions are associated with higher rates of chronic illness, sleep disturbances, and health care utilization when compared to rates among individuals with no diagnosis of personality disorder.^14^,^15^ Evidence shows that ASPD and BPD are related, and that they are serious psychiatric disorders associated with significant consequences, including consequences undergirded by poor emotional and behavioral control (e.g., excessive alcohol use), making the disorders likely to co-occur with AUD.

### Diagnosis limitations and considerations

Because the literature on co-occurrence is largely based on categorical diagnoses, the limitations and biases of the current diagnosis classification system for personality disorders should be considered. A few well-documented limitations include lack of coverage of an individual’s presenting concerns within the existing personality disorders, an arbitrary number of symptoms required for a diagnosis, large variation of presentation and symptoms within each personality disorder, and high co-occurrence of personality disorder categories.^7^ Although substantial evidence supports dimensional as opposed to categorical conceptualizations of personality disorders, such as the five-factor model and the DSM-5 alternative model for personality disorders,^6^ the current exploration of co-occurrence inherently relies on categorical diagnoses.^16^

Consequently, some apparent co-occurrence may be misleading because of overlapping features and aspects of diagnostic bias. Moreover, subthreshold levels of alcohol or personality pathology, such as binge drinking and impulsivity, which are not diagnostic categories, may co-occur before co-occurring alcohol and personality disorders can be detected. Thus, an association between personality disorders and AUD may manifest before formal diagnoses of either condition and may occur at varying levels of pathology. These factors should be considered when examining the conceptualization and diagnosis of co-occurring AUD and personality disorders.

## Epidemiology of Co-Occurring AUD and Personality Disorders

Data from large epidemiological studies of psychopathology highlight the intertwined nature of AUD and personality disorders. In the National Epidemiologic Survey on Alcohol and Related Conditions (NESARC), which was a large, population-based study, 42% of participants who met the diagnostic criteria for any personality disorder also met the criteria for DSM-IV alcohol dependence.^10^ Diagnostic co-occurrence tended to be most pronounced for Cluster B personality disorders, particularly ASPD and BPD, which are characterized by disinhibited and antagonistic forms of externalizing traits and behaviors. Recent reviews have indicated that of those individuals who met diagnostic criteria for BPD, 46% to 49% also met diagnostic criteria for current AUD, and 59% met diagnostic criteria for lifetime AUD.^17^ The prevalence of AUD among those diagnosed with ASPD was about 68%.^18^ Among the general population or clinical samples of individuals with a current diagnosis of AUD or alcohol dependence, the prevalence of a BPD diagnosis was approximately 12% to 17%.^17^ Among individuals with an AUD diagnosis, especially clinical samples, ASPD diagnoses were slightly more prevalent than BPD diagnoses, ranging from 19% to 22%.^18^ Overall, AUD and ASPD and BPD overlap to a high degree.

Nevertheless, it is important to consider co-occurrence estimates in the context of their sampling limitations and interpretive challenges. For instance, many studies that establish populationwide estimates are cross-sectional, which precludes investigating the temporal relations among onset of AUD and personality disorders. Moreover, epidemiological data tend to rely on retrospective self-reports and lifetime diagnoses, which may be influenced by an individual’s current emotional state (e.g., momentary affect) and general personality traits (e.g., level of negative emotionality).

In addition, when assessing for AUD, interviewers ask about the various consequences of alcohol use. In practice, establishing alcohol as a cause or contributor to a criterion (e.g., hazardous use) can be extremely challenging, but the assumption that alcohol played a causal or consequential role is often the default.^19^ For example, if an individual routinely drinks while driving, is this behavior best understood as caused by AUD or by a more general pattern of rule-breaking and risky behavior? Therefore, some ostensible co-occurrence could be due to imprecision in the diagnostic criteria and how those criteria are assessed.

## Explanations and Models of Co-Occurrence

Relevant to developing effective treatment and prevention are the mechanisms responsible for co-occurring AUD and personality disorders, that is, how or why personality disorders relate to AUDs. Explanations or models of co-occurring AUD and ASPD or BPD include common third-variable (e.g., trait) explanations and causal (e.g., AUD leads to personality disorder or personality disorder leads to AUD) explanations.

### Trait explanations

Meta-analytic research suggests that personality disorders can be conceptualized as combinations, or even configurations, of extreme variants of general personality traits, which often are based on or correspond with the five-factor model.^8^ The five-factor model encompasses the broad personality domains of neuroticism, extraversion, openness to experience, agreeableness, and conscientiousness, each of which includes narrower traits, termed “facets.” Five-factor model domains and facets are dimensional, such that variability in personality lies on a continuum, with each pole reflecting an extreme of a basic trait. For simplicity, the two poles are described as high and low. For example, social cooperativeness and affiliation reflect high agreeableness, which is the opposite pole of antagonism. ASPD and BPD reflect low levels of agreeableness and conscientiousness and high levels of antagonism and impulsivity, respectively. ASPD and BPD have key associations with neuroticism and extraversion, although the personality trait associations are different for each disorder. BPD is characterized by high levels of neuroticism, whereas ASPD is not robustly associated with neuroticism but is characterized by high levels of two of neuroticism’s facets: anger and impulsiveness. ASPD is characterized by high levels of the excitement-seeking facet of extraversion, whereas BPD is characterized by low levels of the warmth and positive emotionality facets of extraversion.

From a trait perspective, BPD and ASPD tend to relate similarly to AUD. This similarity can be explained by their overlapping profiles of general personality traits, particularly antagonism and impulsivity (disinhibition).^8^ Although AUD often is conceptualized as an episodic condition rather than a chronic (trait-like) condition, it is increasingly apparent that AUD is related to several personality traits, and that these traits are similar to the traits that undergird ASPD, BPD, and other psychopathology in general. Trull and Sher first established that alcohol abuse and dependence were characterized by high levels of neuroticism and low levels of agreeableness and conscientiousness.^20^ Even with no diagnosis of AUD, features or patterns of alcohol use (e.g., ever using alcohol, quantity of alcohol use, and problematic use of alcohol) have been characterized by the same general personality traits (e.g., low conscientiousness).^21^

Of note, typologies for AUD have shown similar patterns of personality dimensions. Cloninger conceptualized two subtypes of AUD.^22^ Type I had later onset (after age 25) and was associated with more anxious rather than impulsive features. Type II was more common in men and represented individuals who had early onset of alcohol use and frequent aggressive behaviors or arrests. Cloninger examined Type II AUD^22^ and ASPD^23^ separately and posited that they both had high novelty-seeking, low harm avoidance, and low reward dependence. This literature converges evidence that AUD on one hand and BPD and ASPD on the other have comparable relationships with general personality traits. Personality traits associated with aggressive, impulsive, and neurotic tendencies coalesce into the trait complexes of ASPD and BPD. These same trait complexes may contribute to a broad swath of externalizing forms of psychopathology, including alcohol and other substance misuse, risky sex, and other antisocial behavior.^24^,^25^

### Developmental explanations

Adolescence and emerging adulthood are crucial developmental periods for understanding the sources and trajectory of AUD. In addition to being a period of heightened alcohol use,^26^ adolescence tends to be associated with increased independence and acquisition of adult roles, exploration, and reward-seeking, as well as heightened levels of impulsivity, sensation-seeking, and, to a lesser extent, neuroticism.^27^ Declines in alcohol use and reductions in personality trait levels across development have been called “maturing out”^28^ and the “maturity principle,”^29^ respectively. For example, late adolescence and emerging adulthood are associated with heightened prevalence of alcohol use and associated problems, the risk for which tends to decline with age. Although personality traits are believed to reflect a person’s stable, internal disposition,^30^ the transition from emerging to young adulthood is associated with normative changes in personality that reflect development toward psychological maturity, such as increases in emotional stability, self-control, and affiliation, and a shift to adult roles, such as committed relationships and parenthood.^27^

Researchers have empirically linked these developmental changes in personality and alcohol use.^31^–^33^ Specifically, changes in impulsivity, neuroticism, and problematic alcohol use tend to correlate. Across adolescence and early adulthood, individuals with steeper declines in impulsivity and neuroticism demonstrated steeper declines in problematic alcohol use.^33^ Individuals with a less substantial decline (or even an increase) in impulsivity and neuroticism had either increases, or smaller decreases, in problematic alcohol use. In the same vein, increases in risk-taking behavior across development are associated with increases in alcohol use among adolescents.^34^,^35^ Still, there are individual differences in these general developmental trends, and some research suggests that personality may moderate AUD trajectories such that individuals who exhibit more impulsivity and neuroticism are more likely to experience more severe or chronic problems with alcohol. Relatedly, other research suggests variability in the developmental course of personality and alcohol use. Some individuals do not exhibit the maturity principle or mature out of alcohol use and instead exhibit chronic and stable alcohol, emotional, and behavioral control issues.^36^,^37^

### Causal models

At least four major co-occurrence models, each of which contains different assumptions, explain how AUD relates to ASPD and BPD: the predisposition (or vulnerability) model, the complication (or scar) model, the exacerbation model, and the spectrum model.^38^ The predisposition model purports that existing personality disorder elicits environmental responses, such as interpersonal or occupational problems, that provoke the onset of AUD. The temporal relationship between AUD and ASPD or BPD is reversed in the complication model, whereby AUD “scars” an individual’s personality. For instance, neuroadaptation due to excessive alcohol consumption across time might result in increased impulsivity or negative emotionality. The exacerbation model purports that ASPD and BPD add to or modify the manifestation, course, or expression of AUD, resulting in a distinctive AUD symptom profile. For instance, the presence of ASPD or BPD might increase the longevity of AUD or the extent of impairment. The spectrum model posits that the two disorders share common etiology.

Unfortunately, there is a relative paucity of empirical data for comparing these causal models. Existing data tend to support the predisposition model, in which the personality traits that undergird ASPD or BPD, particularly impulsivity, novelty-seeking, and neuroticism, tend to predict later alcohol problems, including AUD diagnosis^39^ and onset.^40^ Tracing the prospective, longitudinal relationships between impulsivity, neuroticism, and AUD across adolescence, Elkins and colleagues demonstrated that, after accounting for preexisting AUD, impulsivity and negative emotionality uniquely predicted new onset of AUD at age 20 after a baseline at age 17.^40^

Still other research suggests that personality may contribute to AUD by means of “niche-picking,” whereby those with higher levels of certain personality traits select into high-risk environments for AUD. Park and colleagues found that undergraduates who scored highly on extraversion, despite not drinking heavily before college, were more likely to enter into the Greek system and thus were at increased risk for alcohol problems later in college.^41^ Novelty-seeking (a facet of extraversion) also has been shown to have a proximal association with alcohol use, such that enhancement motives for drinking (to “get high” or enhance positive affect) were associated with sensation-seeking.^42^ Together, these findings suggest that traits associated with ASPD and BPD, namely impulsivity and negative emotionality, appear to reflect broad liability for precocious alcohol use and AUD. Other traits associated with ASPD, namely novelty-seeking, tend to be associated with AUD both directly and indirectly by influencing selection into high-risk environments and motives for drinking.

The exacerbation model has some limited support, in that individuals with higher levels of outgoingness, impulsivity, aggression, and antisociality have been shown to be more likely to experience reinforcing, stress-dampening effects of alcohol.^43^ The complication model also has some limited support, as demonstrated by research in which chronic, heavy-drinking adolescents exhibited short-term (1 year) increases in impulsive behavior.^35^ Research also has implicated alcohol use as a predictor of aggressive and violent behavior.^24^

Of note, the temporal relatedness of alcohol use to changes in personality is relevant, such that “proximal, but not necessarily distal, alcohol use influences subsequent changes in personality.”^44^^(p363)^ Barnes wrote about the directionality of these relationships, noting that neuroticism tended to increase from “prealcoholic” to “clinical alcoholism,” suggesting that such a change in personality may be a result of heavy or chronic drinking.^45^

The increase in neuroticism as alcohol use progresses aligns with neurobiological models of addiction, such as the allostatic model. This model posits that as addiction and compulsion for a substance progresses, negative affect increases in the absence of the substance, thereby contributing to substance use as negative reinforcement and becoming a continuing cyclical process.^46^ The result is progressive allostatic changes of less positive and more negative mood. The persistence and reversibility of such presumed allostatic effects in the absence of continued heavy drinking is unclear.^45^ Together, these findings highlight the intertwined, bidirectional connections between AUD and personality disorders, which likely cannot be described by one causal model.

The predisposition, complication, and exacerbation models presume independent etiology and onset of AUD and personality disorders. The spectrum model, in contrast, contains two major assumptions: Personality disorders and AUD are not distinct and rise, at least in part, from a set of common etiological factors. In addition, each disorder exists on a continuum or comprises multiple components along a continuum, ranging from subclinical traits to full-blown psychopathology. This model has received considerable support and also has historical roots. Cloninger first proposed that personality mediated genetic risk for AUD,^23^ a theory that Slutske and colleagues later instantiated empirically.^47^ Using a multivariate behavioral genetic twin design, these researchers found that the genetic variance associated with the broad trait of behavioral undercontrol, which included impulsivity, novelty-seeking, and aggression, accounted for 40% of the genetic variance in alcohol dependence. These findings highlight the notion that the overlap of impulsivity and AUD originates from shared genetic mechanisms. Other work has demonstrated the same for AUD and BPD.^48^ This shared genetic mechanism appears to give rise to externalizing behavior and psychopathology generally,^25^ including AUD, other substance use disorder (SUD), conduct disorder, and antisocial behavior, rather than to impulsivity and AUD specifically.

These findings align with burgeoning evidence that internalizing and externalizing are two broad, heritable spectra of psychopathology. Internalizing is characterized by elevated negative emotionality, and externalizing is characterized by behavioral undercontrol and novelty-seeking. These two spectra are responsible for well-documented co-occurrence of psychiatric conditions that share phenomenological similarities.^49^,^50^

Contemporary taxonomies organize psychopathology dimensionally and hierarchically, with signs and symptoms of psychiatric conditions at the bottom of the hierarchy and externalizing and internalizing toward the top.^51^ Much research places AUD, ASPD, and BPD squarely within externalizing. Externalizing can be broken down into disinhibited and antagonistic forms. Disinhibited externalizing comprises all substance-related disorders, whereas antagonistic externalizing comprises BPD as well as narcissistic, histrionic, and paranoid personality disorders. Notably, an antisocial behavior subfactor is believed to contribute to both the disinhibited and antagonistic externalizing subspectra and includes ASPD, conduct disorder, oppositional defiant disorder, attention deficit hyperactivity disorder, and intermittent explosive disorder. Some research suggests that BPD contributes to both externalizing and internalizing spectra,^52^ although this possibility warrants more research attention.

Tully and Iacono proposed a hierarchical common liabilities model, which suggests that disorders (e.g., SUD and ASPD) that load onto the same psychopathology spectrum (e.g., externalizing) share common etiologic mechanisms.^50^ As noted previously, a significant amount of evidence demonstrates that genes influence the covariation among disorders within externalizing and internalizing spectra, likely because of the common neurobiological mechanisms within each spectrum. These researchers offered that neurobiological mechanisms responsible for behavioral control and negative emotionality give rise to externalizing and internalizing, respectively, and likely are responsible for the co-occurrence among AUD, ASPD, and BPD. Specific genetic and other neurobiological mechanisms responsible for the development of AUD, ASPD, and BPD remain elusive. Further research is needed to identify more specific neurobiological mechanisms and biologically based endophenotypes implicated in the covariation among AUD, ASPD, and BPD, as well as those that are unique to each condition.^53^

Closely aligned to the spectrum perspective is the notion that AUD is heterogeneous and has two or more subtypes, each one associated with a different spectrum.^54^,^55^ A number of these subtypes, such as Knight’s “essential” type,^54^ Babor’s Type B,^55^ and Cloninger’s Type II,^22^ are characterized by early onset and antisocial features. Thus, a relevant consideration is the possibility that the apparent co-occurrence between AUD and ASPD, for example, could be viewed as a subtype of AUD associated with the externalizing spectrum. Other subtypes, such as Knight’s “reactive alcoholism,” Babor’s Type A, and Cloninger’s Type I, are associated more with the internalizing spectrum. The subtyping literature highlights that the phenomenon of co-occurrence need not be viewed as the overlap of two relatively homogeneous conditions but could represent a single, relatively homogeneous, subtype of a heterogeneous condition.

## Prognosis and Course

The course of AUD has much variation, with some cases limited to a specific period of time, others showing a relapsing and remitting pattern, and still others showing a persistent, chronic pattern.^56^ Given the chronic nature of personality disorders, it seems likely that the presence of a co-occurring personality disorder would be associated with a more pernicious course of AUD. Relatively little research has used community-based samples to examine the course of AUD and personality disorders. However, existing data suggest co-occurring personality disorders augur poor prognoses. For example, in a general population sample, ASPD and BPD were significantly associated with persistence of alcohol dependence.^57^

Few in-depth investigations focus on the course of co-occurring AUD and ASPD. One study investigated the prevalence and course of SUD, including AUD, in a treatment-seeking sample that included a small number (*n* = 54) of individuals diagnosed with ASPD and a comparison sample (*n* = 552) of individuals with no ASPD diagnosis.^58^ The investigators found that individuals diagnosed with ASPD started drinking alcohol at younger ages. However, AUD diagnosis and indicators of course (i.e., years of alcohol use, days of alcohol use in the past year, and days of abstinence) were not significantly different between the ASPD and non-ASPD groups.

A prospective, 10-year study focused on the course of BPD in a clinical sample and demonstrated a few major themes relevant to the course of SUD, including alcohol abuse and dependence.^59^ The study included two groups of participants: those diagnosed with BPD and those diagnosed with another personality disorder. First, diagnoses of alcohol abuse and dependence were more common among participants who were diagnosed with BPD when compared with participants diagnosed with another personality disorder. Second, the prevalence of alcohol disorders similarly decreased over time for both groups, but it remained more common among those diagnosed with BPD.

The course of alcohol and substance disorders was examined more closely within the BPD group. The findings indicated that a vast majority (about 90%) of participants diagnosed with BPD had a remission of alcohol abuse or dependence by the 10-year follow-up.^59^ Further, participants with BPD were more likely to experience remission than recurrences of use, and individuals who had BPD but no alcohol diagnosis at baseline were unlikely to develop an alcohol-related diagnosis during the study. Although this was not a treatment-specific study, the participants were recruited from inpatient samples and were in treatment for most of the study period.

In a review of treatment outcomes for individuals with co-occurring AUD and ASPD, Newton-Howes and colleagues concluded that alcohol outcomes and psychosocial functioning improved for those who stayed in treatment, although attrition was high.^60^ The prognosis of co-occurring AUD and BPD is complex and difficult to disentangle given the varied pathways of each disorder. Intensive longitudinal studies are critical to assess variations in course and prognosis and can potentially provide indicators of co-occurrence and severity. Additional research in this area is needed.

## Treatment

Clinical approaches to and research on treatment for personality disorders and SUD (including AUD) have often been tackled from a silo approach, such that one condition (e.g., addiction) is addressed separately from other psychological symptoms and disorders. Addressing personality disorders and SUD independently may be necessary in the clinical realm because of active substance use or threats of relapse thwarting treatment progress. Also, this approach may be necessary for research trials to maximize internal validity.

Depending on the severity of AUD, the detoxification period may first be necessary for the most accurate assessment of mood and personality. For example, increased irritability, anxiety, and low mood may be present primarily during heavy use or during withdrawal and may resolve if substance induced.^2^,^46^ Assessment of affective symptoms after withdrawal or detoxification, incorporating known information about premorbid emotional and behavioral functioning when available, may help with diagnosis decisions and may serve to disentangle substance use from symptoms that may be associated with other disorders. However, some individuals do not receive treatment following detoxifications, as it is estimated that approximately 50% of detoxifications are followed by other treatment.^61^

Although co-occurring AUD or SUD and personality disorders understandably can make the assessment and intervention process challenging, it may be unrealistic to require that treatment focus on only one aspect at a time (e.g., target only substance use and then treat the personality disorder). For co-occurring AUD and BPD, a number of complications may arise, such as suicidal thoughts or behavior associated with the personality disorder, potentially undermining the ability to continue with AUD treatment. Thus, it may not always be possible or ideal to treat only the AUD or personality disorder and then proceed to treat the co-occurring disorder. These complexities are evident throughout the research literature, as few studies specifically examine co-occurring conditions.

Although treatments have been developed or adapted for AUD, SUD, BPD, and ASPD, there is limited empirical support for these treatments among samples of individuals diagnosed with AUD and co-occurring ASPD or BPD. Treatment research involving those with AUD and psychiatric disorders other than personality disorders also is limited, highlighting a major gap in empirical and intervention fields.^62^ However, studies examining various disorder-specific treatments may be useful for treating the co-occurring disorders. It should be noted that a number of treatments may be effective for AUD and ASPD or BPD, but they have not been established as efficacious because of limited trials, small samples, or a broad focus on SUD or outcomes rather than AUD.^63^ Regardless, research in which SUD is the focus may provide a starting point for further treatment research on alcohol use and AUD in the context of BPD and ASPD. (See Table 1 for brief descriptions of the treatments discussed in this article.)

### Psychosocial treatments

There is modest support for treatments that show reductions in substance use while primarily treating BPD (i.e., dialectical behavior therapy, dynamic deconstructive psychotherapy, and dual-focused schema therapy) or while treating SUD in the context of BPD.^63^,^76^ For example, one study examined dialectical behavior therapy for SUD and included medication assistance (e.g., replacing opiates with methadone) in the initial phases of treatment, called “transitional maintenance.”^64^ The investigators reported that at the end of treatment and at a 16-month follow-up, this treatment was more effective at reducing substance use than treatment as usual.

Other studies have found dialectical behavior therapy to be as effective at treating BPD symptoms for those with BPD and SUD as it is for participants with no SUD.^77^ However, for the reduction of substance-related symptoms, no difference was found between the dialectical behavior therapy group and the treatment as usual group.^77^ Although dialectical behavior therapy is primarily used for BPD, it was found to be acceptable in a clinical trial intended to treat men with both BPD and ASPD, most of whom also reported substance use.^78^ Rates of alcohol and substance use did not change substantially in this trial, however. A review examining effective treatments for BPD determined that other treatments, such as mentalization-based therapy, showed promise, although the small number of studies limited the strength of possible recommendations.^79^

Effective treatments for ASPD are limited because few trials with sufficient evidence have been identified.^80^ ASPD treatments showing promise, such as treatment with contingency management, often were originally developed for SUDs, further highlighting the possibility of a common thread across interventions for co-occurring AUD and ASPD or BPD.

As noted by Garofalo and Wright, treatment approaches based on transdiagnostic constructs such as neuroticism and disinhibition may target changes in the constructs.^24^ Transdiagnostic factors, which have been described as “psychological constructs that are observed across a range of disorders” and “functionally causal mechanisms that inform the development of classes of disorders,” align with a dimensional approach to both understanding and treating psychopathology.^81^^(p135)^ Some treatment packages that use a transdiagnostic approach are acceptance and commitment therapy,^71^ dialectical behavior therapy,^64^ and the unified protocol.^72^ Through various treatments and across an array of disorders, including BPD and SUD, research has supported changes related to transdiagnostic constructs, such as increases in emotion regulation.^82^ In addition, indirect evidence supporting transdiagnostic approaches comes from research on personality and alcohol, which has revealed that using alcohol to cope with negative emotions mediates the association between personality traits, such as neuroticism and impulsivity, and reported alcohol problems.^83^

The integration of relevant treatment components such as emotion regulation skills, as opposed to stand-alone, single-disorder treatment, is highly compatible with transdiagnostic approaches. For example, contingency management, an effective treatment for AUD that uses behavioral principles to decrease ineffective and increase effective behaviors, has been incorporated into treatment for other disorders, such as dialectical behavior therapy.^70^ Integrated treatment for personality disorders proposes using key treatment components from multiple therapies and developing a treatment adapted to a patient’s needs.^74^ This approach inherently integrates key transdiagnostic components such as emotion regulation.

Research specific to co-occurring SUD and personality disorders (not exclusively ASPD and BPD) has concluded that using evidence-based strategies across therapies (e.g., combining contingency management with pharmacotherapy) tends to be most effective.^84^ Research on AUD treatment has suggested that targeting specific traits, such as impulsiveness, using a matched treatment approach may effectively reduce alcohol use.^85^ Mindfulness and modification therapy, which is another transdiagnostic treatment that targets behavioral dysregulation, has been shown to be related to decreased alcohol use and aggression among voluntary and court-ordered participants.^75^ Collectively, the research suggests that identifying transdiagnostic features and treating conditions using evidence-supported treatment components that target those features may be a useful approach for treating co-occurring AUD and personality disorders.

Important to note is attrition during treatment for co-occurring AUD or SUD and personality disorders (e.g., 40% in a sample of SUD and BPD), and some evidence shows higher dropout rates for participants who had AUD and a personality disorder, as compared to those with AUD and no personality disorder.^60^,^64^ This attrition is not surprising given that this population faces many challenges and complexities with the presenting problem and related to the broader environment and context. However, some studies have pointed to factors and existing strategies that may improve retention rates, such as making treatment enrollment contingent on predetermined attendance rules and establishing strong therapeutic relationships.^64^ Other research has called for a focus on improving dual-diagnosis treatments and retention strategies for people with AUD.^60^

### Pharmacological interventions

Comprehensive treatment for people with co-occurring AUD and ASPD or BPD often adopts a multifaceted approach using psychosocial and pharmacological interventions, including medication-assisted treatment for AUD and for BPD. Treatment for AUD may include acamprosate, naltrexone, disulfiram, or off-label medications such as topiramate,^86^ and treatment for BPD may include naltrexone or topiramate.^87^,^88^ This review focuses on studies of personality disorders and AUD outcomes and is organized by class of medication (i.e., alcohol-specific medications, anticonvulsants, and psychoactive drugs).

An investigation of the effectiveness of medications among individuals with alcohol dependence found that treatment with naltrexone, naltrexone plus disulfiram, or disulfiram plus placebo was just as effective for alcohol use outcomes among individuals who had co-occurring BPD or ASPD as it was among those with no ASPD or BPD.^89^ In another study, Rohsenow and colleagues identified that the presence of antisocial traits was associated with increased effectiveness of naltrexone when compared to placebo.^90^

Before discussing pharmacotherapy for personality disorders, it should be noted that no medications for ASPD or BPD have been approved by the U.S. Food and Drug Administration. Further, no clinical trials have directly examined the efficacy of medications for people with co-occurring AUD and ASPD or BPD. Most studies have focused on one medication that targets similar mechanisms (e.g., impulsivity) across co-occurring conditions.

Research supporting specific pharmacotherapy for BPD is mixed, largely because the quality and quantity of studies provide insufficient evidence to evaluate efficacy.^91^ Although the evidence regarding pharmacotherapy approaches for BPD is equivocal, certain medications, such as mood stabilizers and antipsychotics, matched to specific symptom presentations, such as affective lability, may show improvement for BPD symptoms, whereas selective serotonin reuptake inhibitors (SSRIs) demonstrate little to no efficacy.^87^ Similarly, studies have preliminarily supported use of naltrexone for symptoms in the impulsive behavior domain and have reported reductions in self-injurious thoughts and behaviors.^87^,^88^ The general recommendation is to use psychotherapy as the primary treatment with pharmacotherapy as an adjunctive treatment, since the efficacy of specific medications for BPD is not currently robust. Regarding ASPD, little evidence supports pharmacotherapy, and medications are often used to treat symptoms but not as a stand-alone treatment.^92^

Anticonvulsants such as topiramate and lamotrigine and atypical, second-generation antipsychotics such as olanzapine have been investigated for the treatment of AUD and BPD. Topiramate has been identified as a possible off-label medication for AUD and BPD separately, suggesting a mechanism of action (of increased inhibitory control) applicable to both conditions.^93^ A review of the medications for co-occurring AUD and BPD noted that topiramate was associated with fewer drinking days for participants who had AUD and with decreased anger intensity and reactions for those who had BPD.^63^ In addition, topiramate and lamotrigine have demonstrated some effectiveness for decreasing craving, and lamotrigine has been associated with a decrease in impulsivity and anger symptoms of BPD.^63^

The atypical antipsychotics aripiprazole and olanzapine have been associated with impulsivity changes in BPD.^94^,^95^ The effect of atypical antipsychotics on alcohol-related outcomes is mixed. An inconsistent effect for outcomes such as craving or abstinence has been reported across studies, and some research has suggested that genetic influences may act as primary moderators.^96^,^97^

The literature on antidepressants has demonstrated mixed results across studies and conditions. As previously mentioned, SSRIs generally have been ineffective in the treatment of BPD. On the other hand, research investigating AUD and ASPD has found more promising results. For instance, one study concluded that people with AUD and ASPD who also had another mood disorder benefited from antidepressants, whereas those with no additional mood disorder did not.^98^ In a review of pharmacotherapy for ASPD, the tricyclic antidepressant nortriptyline was identified as one of the medications that was superior to placebo on at least one alcohol-related outcome (i.e., drinking days).^92^ However, only one study reported this result, and several other outcomes, such as patient drinking ratings and craving, did not differ between the nortriptyline and placebo groups.^92^ As with the atypical antipsychotics, antidepressants have been associated with different pharmacological outcomes across the traditional alcohol typologies. For individuals in the Type A typology group compared with those in the Type B group, the SSRI sertraline was more effective for the outcomes of fewer drinking days, time to relapse, and continuous abstinence period.^99^

### Considerations and future directions for treatment

ASPD and BPD are complex and heterogeneous disorders often accompanied by other disorders, such as anxiety or depression. Therefore, as with psychosocial approaches, pharmacotherapy has focused on transdiagnostic dimensions or assumed neurophysiology rather than diagnosis categories for treatment of these disorders.^100^ This focus has led to the investigation of medications specific to affective dysregulation or impulsive behavioral dysregulation instead of medications specific to a diagnosis.

Other treatment complexities include determining level of care based on severity of presentation and addressing barriers to accessible care. For individuals with severe AUD, inpatient or detoxification treatment may be a necessary component of treatment. For individuals with BPD, hospitalization or specific safety measures may be necessary if suicide is a risk. For those with ASPD, incarceration or other related limitations may be barriers to treatment. When any of these disorders occur independently or simultaneously, the risks of addiction, intentional or accidental overdose, and self-harm are heightened and may affect the course of treatment, particularly pharmacotherapy decisions.

Stepped care is an approach that can potentially help navigate the complex and evolving nature of co-occurring AUD and ASPD or BPD. Stepped care, or continuing care, has been associated with positive outcomes and longer treatment engagement for individuals with AUD or SUD.^101^ Stepped care is an adaptive approach, evolving as the patient’s needs change over time. For example, intensive in-person treatment may be necessary at times, whereas other modes of treatment with varying levels of intensity, such as telephone-based care or medication, may be more appropriate over the period of treatment. A flexible treatment team is necessary for a stepped care approach to work effectively. Time in treatment has been positively associated with better outcomes for people who have been diagnosed with co-occurring AUD and other psychopathology,^84^ further highlighting the potential utility of stepped care approaches for these co-occurring conditions.

In conclusion, future research investigating pharmacotherapies specific to co-occurring conditions is needed. The extant research, often limited to a few studies per finding, generally concludes:

- Pharmacotherapies for AUD do not produce different outcomes for individuals with a co-occurring personality disorder.
- Some anticonvulsants and atypical antipsychotics may be useful for the treatment of AUD, BPD, and their co-occurrence.
- Research is mixed on the effectiveness of antidepressants for ASPD alone and for co-occurring AUD and ASPD, and effectiveness often depends on important moderating variables.

Evidence-based treatments for co-occurring AUD and personality disorders, in addition to realistic implementation and dissemination strategies that accommodate the treatments to these multifaceted disorders, need to be explored further.

## Conclusion and Future Research

Existing research on ASPD and BPD has important implications for AUD, likely because the conditions have overlapping symptoms, personality correlates, course, and etiology. Research examining shared mechanisms can contribute to both prevention and targeted intervention efforts. In addition, using new and advanced methodological approaches to assess risk factors and precursors to misuse or relapse can advance understanding of mechanisms that contribute to initial and continued use along the developmental course.^26^

Key aspects of these disorders, such as affect disturbance, reflect volatility. Momentary changes in affect may be challenging to recall or assess using traditional methodological approaches such as asking individuals to rate their mood from a week ago. For example, craving and affect are episodic and may be assessed more accurately when they occur with natural cues. Precise assessment of such symptoms or constructs is relevant to diagnosis, because a comprehensive assessment of important criteria across relevant contexts can provide a full and more accurate picture of the individual’s presenting concerns and symptoms. Research incorporating methodological approaches, such as ambulatory assessment and ecological momentary assessment, to assess mood and craving in the moment can resolve critical within-person patterns of response to evocative cues, allowing for a more nuanced and individual evaluation of associations between the behaviors (e.g., drinking) and traits (e.g., impulsivity) commonly related to personality disorders. These methods can facilitate the assessment of an individual’s experience (e.g., mood and behaviors) in the moment.

Future research should also continue to focus on assessing and implementing the best methods, times, and places for providing treatment to individuals with co-occurring AUD and ASPD or BPD. For example, individuals with both AUD and a personality disorder tend to seek substance-specific treatment later than those with only AUD, although, on average, they use substances earlier, have greater impairment, and have shorter time to relapse.^102^ Research must also address:

- Screening (where and when people get referred to treatment)
- Barriers to treatment entry (factors that influence failure to enter treatment)
- Identification of treatment approaches for co-occurring conditions
- Dissemination and implementation of effective treatment approaches

All three disorders have many similarities, including impulsivity and negative affect, externalizing correlates, and a likely potential for serious consequences and negative outcomes. Nevertheless, the ability to reach people diagnosed with these conditions and to treat them successfully is lacking in many ways. Individuals diagnosed with co-occurring AUD and ASPD, BPD, or another personality disorder clearly have an influential presence across health and legal systems.^12^,^15^ However, people diagnosed with AUD alone have a surprisingly low treatment-seeking rate.^62^ In the National Comorbidity Survey, results specific to treatment-seeking behaviors among individuals with co-occurring AUD and a psychiatric condition indicated that this population was more likely to receive specialty mental health treatment not focused on substance use (41%) than substance-specific interventions (16%).^62^

The contrast between patterns of treatment-seeking behaviors is stark for people diagnosed with AUD alone versus those diagnosed with co-occurring conditions. Further understanding of the barriers to treatment for those with co-occurring conditions may provide points of change that positively influence the consumer’s ability to access care that targets relevant transdiagnostic factors. Hopefully, as more investigators focus on the common factors underlying these conditions, newer assessment and treatment approaches can be developed, evaluated, and ultimately disseminated to settings and clinicians that serve these individuals.

## Figures and Tables

**Table 1:** Treatment Descriptions

Treatment	Key Concepts
Dialectical Behavior Therapy for SUD^64^	Uses primarily behavioral approaches to target problematic behaviors organized within a predetermined hierarchy: life-threatening behaviors, behaviors that interfere with treatment, and behaviors that interfere with quality of life. Targets substance use as the top behavior within the quality-of-life level of the hierarchy. Includes skills training in four domains: mindfulness, emotion regulation, distress tolerance, and interpersonal effectiveness. Includes 12 months of weekly individual therapy and group skills training, telephone coaching, and therapist consultation. Emphasizes attachment strategies and dialectical abstinence. Targets BPD and AUD simultaneously.
Dynamic Deconstructive Psychotherapy^65^	Includes weekly individual therapy for 12 months. Emphasizes alliance building, emotion identification, polarization awareness, judgment awareness and modification, and distance from idealizing fantasies. Targets AUD and BPD simultaneously.
Dual-Focused Schema Therapy^66^	Includes 6 months of individual and group therapies. Emphasizes relapse prevention, stimulus control, interpersonal and emotion regulation skills, coping with craving, and identification and obstruction of maladaptive schemas. Addresses substance use as a coping mechanism for emotions and conflicts related to schemas. Targets AUD and BPD simultaneously.
Mentalization-Based Therapy^67^	Uses psychodynamic-oriented treatment in group and individual formats. Emphasizes improvement of mentalization within a safe, collaborative, and attached therapy relationship and focuses on internal states of self and others, with a goal of improving interpersonal relatedness, emotion regulation, and identity.
Metacognitive Treatment^68^,^69^	Emphasizes metacognitive mastery, which is the “ability to use knowledge about mental states of self and others to cope with distress and solve social problems.”^6^^(p22)^ Targets the cognitive attentional syndrome to modify unhelpful thinking patterns.
Contingency Management^70^	Uses behavioral economics and operant conditioning principles to modify behaviors. Emphasizes the use of reinforcements and consequences to increase desired (e.g., abstinence) and decrease undesired (e.g., substance use) behaviors.
Acceptance and Commitment Therapy^71^	Emphasizes acceptance, values, and psychological flexibility through approaches such as mindfulness, identification of values and congruent living, and thought diffusion. Offers individual and group formats.
Unified Protocol Therapy^72^	Uses transdiagnostic treatment for emotional disorders. Emphasizes emotional and physical awareness, appraisal flexibility, exposure, and emotion-driven behaviors.
Emotion-Regulation Therapy^73^	Uses an acceptance-based approach to emotion regulation and is delivered in group format as an adjunctive treatment. Includes participation in groups focused on improving skills such as, among others, impulse control and increasing awareness of emotions and their functions.
Integrated Therapy^74^	Uses a coordinated, goal-oriented approach integrating evidence-based components of other treatments (e.g., dialectical behavior therapy and cognitive behavioral therapy) and follows a sequential process of therapy stages, beginning with establishing safety. Emphasizes therapeutic relationships, motivation for change, and self-observation.
Mindfulness and Modification Therapy^75^	Includes individual or group transdiagnostic treatment targeting behavioral dysregulation. Emphasizes mindfulness and components of other treatments (e.g., acceptance and commitment therapy and dialectical behavior therapy).

*Note*: This table does not include all the available treatment approaches, and these descriptions are not intended to be comprehensive descriptions of the treatments or their components.

## References

[1] American Psychiatric Association (2000). Diagnostic and Statistical Manual of Mental Disorders.

[2] American Psychiatric Association (2013). Diagnostic and Statistical Manual of Mental Disorders.

[3] Saha TD, Chou S, Grant BF (2006). Toward an alcohol use disorder continuum using item response theory: Results from the National Epidemiologic Survey on Alcohol and Related Conditions. Psychol Med.

[4] World Health Organization (2018). International Statistical Classification of Diseases and Related Health Problems.

[5] Costa PT, McCrae RR (1992). Normal personality assessment in clinical practice: The NEO Personality Inventory. Psychol Assess.

[6] Hopwood CJ, Kotov R, Krueger RF (2018). The time has come for dimensional personality disorder diagnosis. Personal Ment Health.

[7] Trull TJ, Durrett CA (2005). Categorical and dimensional models of personality disorder. Annu Rev Clin Psychol.

[8] Samuel DB, Widiger TA (2008). A meta-analytic review of the relationships between the five-factor model and DSM-IV-TR personality disorders: A facet level analysis. Clin Psychol Rev.

[9] Armstrong JD (1958). The search for the alcoholic personality. Ann Am Acad Polit Soc Sci.

[10] Trull TJ, Jahng S, Tomko RL (2010). Revised NESARC personality disorder diagnoses: Gender, prevalence, and comorbidity with substance dependence disorders. J Pers Disord.

[11] Zimmerman M, Rothschild L, Chelminski I (2005). The prevalence of DSM-IV personality disorders in psychiatric outpatients. Am J Psychiatry.

[12] Moran P (1999). The epidemiology of antisocial personality disorder. Soc Psychiatry Psychiatr Epidemiol.

[13] McCloskey MS, Ammerman BA (2018). Suicidal behavior and aggression-related disorders. Curr Opin Psychol.

[14] Dixon-Gordon KL, Conkey LC, Whalen DJ (2018). Recent advances in understanding physical health problems in personality disorders. Curr Opin Psychol.

[15] Quirk SE, Berk M, Chanen AM (2016). Population prevalence of personality disorder and associations with physical health comorbidities and health care service utilization: A review. Personal Disord.

[16] Lilienfeld SO, Waldman ID, Israel AC (1994). A critical examination of the use of the term and concept of comorbidity in psychopathology research. Clin Psych.

[17] Trull TJ, Freeman LK, Vebares TJ (2018). Borderline personality disorder and substance use disorders: An updated review. Borderline Personal Disord Emot Dysregul.

[18] Trull TJ, Solhan MB, Brown WC, Sher KJ (2016). Substance use disorders and personality disorders. The Oxford Handbook of Substance Use and Substance Use Disorders.

[19] Martin CS, Langenbucher JW, Chung T (2014). Truth or consequences in the diagnosis of substance use disorders. Addiction.

[20] Trull TJ, Sher KJ (1994). Relationship between the five-factor model of personality and Axis I disorders in a nonclinical sample. J Abnorm Psychol.

[21] Lejuez CW, Magidson JF, Mitchell SH (2010). Behavioral and biological indicators of impulsivity in the development of alcohol use, problems, and disorders. Alcohol Clin Exp Res.

[22] Cloninger CR (1987). Neurogenetic adaptive mechanisms in alcoholism. Science.

[23] Cloninger CR (1987). A systematic method for clinical description and classification of personality variants: A proposal. Arch Gen Psychiatry.

[24] Garofalo C, Wright AGC (2017). Alcohol abuse, personality disorders, and aggression: The quest for a common underlying mechanism. Aggress Violent Behav.

[25] Krueger RF, Markon KE, Patrick CJ (2005). Externalizing psychopathology in adulthood: A dimensional-spectrum conceptualization and its implications for DSM-V. J Abnorm Psychol.

[26] Chassin L, Sher KJ, Hussong A (2013). The developmental psychopathology of alcohol use and alcohol disorders: Research achievements and future directions. Dev Psychopathol.

[27] Roberts BW, Walton KE, Viechtbauer W (2006). Patterns of mean-level change in personality traits across the life course: A meta-analysis of longitudinal studies. Psychol Bull.

[28] Littlefield AK, Winograd RP, Miller P (2013). Maturing out. Principles of Addiction: Comprehensive Addictive Behaviors and Disorders.

[29] Caspi A, Roberts BW, Shiner RL (2005). Personality development: Stability and change. Annu Rev Psychol.

[30] McCrae RR, Costa PT, Ostendorf F (2000). Nature over nurture: Temperament, personality, and life span development. J Pers Soc Psychol.

[31] Hicks BM, Durbin CE, Blonigen DM (2012). Relationship between personality change and the onset and course of alcohol dependence in young adulthood. Addiction.

[32] Iacono WG, Malone SM, McGue M (2008). Behavioral disinhibition and the development of early-onset addiction: Common and specific influences. Annu Rev Clin Psychol.

[33] Littlefield AK, Sher KJ, Wood PK (2009). Is “maturing out” of problematic alcohol involvement related to personality change?. J Abnorm Psychol.

[34] MacPherson L, Magidson JF, Reynolds EK (2010). Changes in sensation seeking and risk-taking propensity predict increases in alcohol use among early adolescents. Alcohol Clin Exp Res.

[35] White HR, Marmorstein NR, Crews FT (2011). Associations between heavy drinking and changes in impulsive behavior among adolescent boys. Alcohol Clin Exp Res.

[36] Johnson W, Hicks BM, McGue M (2007). Most of the girls are alright, but some aren’t: Personality trajectory groups from ages 14 to 24 and some associations with outcomes. J Pers Soc Psychol.

[37] Robins RW, Fraley RC, Roberts BW (2001). A longitudinal study of personality change in young adulthood. J Pers.

[38] Krueger RF, Tackett JL (2003). Personality and psychopathology: Working toward the bigger picture. J Pers Disord.

[39] Sher KJ, Trull TJ, Bartholow B, Blane H, Leonard K (1999). Personality and alcoholism: Issues, methods, and etiological processes. Psychological Theories of Drinking and Alcoholism.

[40] Elkins IJ, King SM, McGue M (2006). Personality traits and the development of nicotine, alcohol, and illicit drug disorders: Prospective links from adolescence to young adulthood. J Abnorm Psychol.

[41] Park A, Sher KJ, Wood PK (2009). Dual mechanisms underlying accentuation of risky drinking via fraternity/sorority affiliation: The role of personality, peer norms, and alcohol availability. J Abnorm Psychol.

[42] Cooper ML, Frone MR, Russell M (1995). Drinking to regulate positive and negative emotions: A motivational model of alcohol use. J Pers Soc Psychol.

[43] Sher KJ, Levenson RW (1982). Risk for alcoholism and individual differences in the stress-response-dampening effect of alcohol. J Abnorm Psychol.

[44] Littlefield AK, Sher KJ, Sher KJ (2016). Personality and substance use disorders. The Oxford Handbook of Substance Use and Substance Use Disorders.

[45] Barnes GE (1979). The alcoholic personality: A reanalysis of the literature. J Stud Alcohol.

[46] Koob GF, Le Moal M (2001). Drug addiction, dysregulation of reward, and allostasis. Neuropsychopharmacology.

[47] Slutske WS, Heath AC, Madden PA (2002). Personality and the genetic risk for alcohol dependence. J Abnorm Psychol.

[48] Bornovalova MA, Hicks BM, Iacono WG (2013). Longitudinal twin study of borderline personality disorder traits and substance use in adolescence: Developmental change, reciprocal effects, and genetic and environmental influences. Personal Disord.

[49] Krueger RF, Markon KE (2006). Reinterpreting comorbidity: A model-based approach to understanding and classifying psychopathology. Annu Rev Clin Psychol.

[50] Tully EC, Iacono WG, Sher KJ (2016). An integrative common liabilities model for the comorbidity of substance use disorders with externalizing and internalizing disorders. The Oxford Handbook of Substance Use and Substance Use Disorders.

[51] Kotov R, Krueger RF, Watson D (2017). The Hierarchical Taxonomy of Psychopathology (HiTOP): A dimensional alternative to traditional nosologies. J Abnorm Psychol.

[52] Eaton NR, Krueger RF, Keyes KM (2011). Borderline personality disorder co-morbidity: Relationship to the internalizing-externalizing structure of common mental disorders. Psychol Med.

[53] Kwako LE, Momenan R, Litten RZ (2016). Addictions Neuroclinical Assessment: A neuroscience-based framework for addictive disorders. Biol Psychiatry.

[54] Knight RP (1937). The dynamics and treatment of chronic alcohol addiction. Bull Menninger Clin.

[55] Babor TF, Hofmann M, DelBoca FK (1992). Types of alcoholics, I: Evidence for an empirically derived typology based on indicators of vulnerability and severity. Arch Gen Psychiatry.

[56] Jacob T, Bucholz KK, Sartor CE (2005). Drinking trajectories from adolescence to the mid-forties among alcohol dependent males. J Stud Alcohol.

[57] Hasin DS, Fenton MC, Skodol A (2011). Personality disorders and the 3-year course of alcohol, drug, and nicotine use disorders. Arch Gen Psychiatry.

[58] Westermeyer J, Thuras P (2005). Association of antisocial personality disorder and substance disorder morbidity in a clinical sample. Am J Drug Alcohol Abuse.

[59] Zanarini MC, Frankenbur FR, Weingeroff JL (2011). The course of substance use disorders in patients with borderline personality disorder and Axis II comparison subjects: A 10-year follow-up study. Addiction.

[60] Newton-Howes GM, Foulds JA, Guy NH (2017). Personality disorder and alcohol treatment outcome: Systematic review and meta-analysis. Br J Psychiatry.

[61] Mark TL, Dilonardo JD, Chalk M (2003). Factors associated with the receipt of treatment following detoxification. J Subst Abuse Treat.

[62] Petrakis IL, Gonzalez G, Rosenheck R (2002). Comorbidity of alcoholism and psychiatric disorders: An overview. Alcohol Res Health.

[63] Gianoli MO, Jane JS, O’Brien E (2012). Treatment for comorbid borderline personality disorder and alcohol use disorders: A review of the evidence and future recommendations. Exp Clin Psychopharmacol.

[64] Linehan MM, Schmidt H, Dimeff LA (1999). Dialectical behavior therapy for patients with borderline personality disorder and drug-dependence. Am J Addict.

[65] Gregory RJ, Remen AL (2008). A manual-based psychodynamic therapy for treatment-resistant borderline personality disorder. Psychotherapy.

[66] Ball SA (1998). Manualized treatment for substance abusers with personality disorders: Dual focus schema therapy. Addict Behav.

[67] Bateman AW, Fonagy P (2004). Mentalization-based treatment of BPD. J Pers Disord.

[68] Dimaggio G, Montano A, Popolo R (2015). Metacognitive Interpersonal Therapy for Personality Disorders: A Treatment Manual.

[69] Outcalt J, Dimaggio G, Popolo R (2016). Metacognition moderates the relationship of disturbances in attachment with severity of borderline personality disorder among persons in treatment of substance use disorders. Compr Psychiatry.

[70] Prendergast M, Podus D, Finney J (2006). Contingency management for treatment of substance use disorders: A meta-analysis. Addiction.

[71] Hayes SC, Luoma JB, Bond FW (2006). Acceptance and commitment therapy: Model, processes and outcomes. Behav Res Ther.

[72] Barlow DH, Farchione TJ, Sauer-Zavala S (2017). Unified Protocol for Transdiagnostic Treatment of Emotional Disorders: Therapist Guide.

[73] Gratz KL, Gunderson JG (2006). Preliminary data on an acceptance-based emotion regulation group intervention for deliberate self-harm among women with borderline personality disorder. Behav Ther.

[74] Livesley WJ (2012). Integrated treatment: A conceptual framework for an evidence-based approach to the treatment of personality disorder. J Pers Disord.

[75] Wupperman P, Cohen MG, Haller DL (2015). Mindfulness and modification therapy for behavioral dysregulation: A comparison trial focused on substance use and aggression. J Clin Psychol.

[76] Lee NK, Cameron J, Jenner L (2015). A systematic review of interventions for co-occurring substance use and borderline personality disorders. Drug Alcohol Rev.

[77] van den Bosch LM, Verheul R, Schippers GM (2002). Dialectical behavior therapy of borderline patients with and without substance use problems: Implementation and long-term effects. Addict Behav.

[78] Wetterborg D, Dehlbom P, Långström N (2018). Dialectical behavior therapy for men with borderline personality disorder and antisocial behavior: A clinical trial. J Pers Disord.

[79] Stoffers-Winterling JM, Völlm BA, Rücker G (2012). Psychological therapies for people with borderline personality disorder. Cochrane Database Syst Rev.

[80] Gibbon S, Duggan C, Stoffers J (2010). Psychological interventions for antisocial personality disorder. Cochrane Database Syst Rev.

[81] Sauer-Zavala S, Gutner CA, Farchione TJ (2017). Current definitions of “transdiagnostic” in treatment development: A search for consensus. Behav Ther.

[82] Sloan E, Hall K, Moulding R (2017). Emotion regulation as a transdiagnostic treatment construct across anxiety, depression, substance, eating and borderline personality disorders: A systematic review. Clin Psychol Rev.

[83] Littlefield AK, Sher KJ, Wood PK (2010). Do changes in drinking motives mediate the relation between personality change and “maturing out” of problem drinking?. J Abnorm Psychol.

[84] Kelly TM, Daley DC, Douaihy AB (2012). Treatment of substance abusing patients with comorbid psychiatric disorders. Addict Behav.

[85] Conrod PJ (2016). Personality-targeted interventions for substance use and misuse. Curr Addict Rep.

[86] Jonas DE, Amick HR, Feltner C (2014). Pharmacotherapy for adults with alcohol use disorders in outpatient settings: A systematic review and meta-analysis. JAMA.

[87] Lieb K, Völlm B, Rücker G (2010). Pharmacotherapy for borderline personality disorder: Cochrane systematic review of randomised trials. Br J Psychiatry.

[88] Sonne S, Rubey R, Brady K (1996). Naltrexone treatment of self-injurious thoughts and behaviors. J Nerv Ment Dis.

[89] Ralevski E, Ball S, Nich C (2007). The impact of personality disorders on alcohol-use outcomes in a pharmacotherapy trial for alcohol dependence and comorbid Axis I disorders. Am J Addict.

[90] Rohsenow DJ, Miranda R, McGeary JE (2007). Family history and antisocial traits moderate naltrexone’s effects on heavy drinking in alcoholics. Exp Clin Psychopharmacol.

[91] Stoffers J, Völlm BA, Rücker G (2010). Pharmacological interventions for borderline personality disorder. Cochrane Database Syst Rev.

[92] Khalifa N, Duggan C, Stoffers J (2010). Pharmacological interventions for antisocial personality disorder. Cochrane Database Syst Rev.

[93] Rubio G, Ponce G, Jiménez-Arriero MA (2004). Effects of topiramate in the treatment of alcohol dependence. Pharmacopsychiatry.

[94] Soler J, Pascual JC, Campins J (2005). Double-blind, placebo-controlled study of dialectical behavior therapy plus olanzapine for borderline personality disorder. Am J Psychiatry.

[95] Zanarini MC, Frankenburg FR (2001). Olanzapine treatment of female borderline personality disorder patients: A double-blind, placebo-controlled pilot study. J Clin Psychiatry.

[96] Hutchison KE, Wooden A, Swift RM (2003). Olanzapine reduces craving for alcohol: A DRD4 VNTR polymorphism by pharmacotherapy interaction. Neuropsychopharmacology.

[97] Martinotti G, DiNicola M, Di Giannantonio M (2009). Aripiprazole in the treatment of patients with alcohol dependence: A double-blind comparison trial vs. naltrexone. J Psychopharmacol.

[98] Penick EC, Powell BJ, Campbell J (1996). Pharmacological treatment for antisocial personality disorder alcoholics: A preliminary study. Alcohol Clin Exp Res.

[99] Pettinati HM, Volpicelli JR, Kranzler HR (2000). Sertraline treatment for alcohol dependence: Interactive effects of medication and alcoholic subtype. Alcohol Clin Exp Res.

[100] Soloff PH (1998). Algorithms for pharmacological treatment of personality dimensions: Symptom-specific treatments for cognitive-perceptual, affective, and impulsive-behavioral dysregulation. Bull Menninger Clin.

[101] McKay JR (2006). Continuing care in the treatment of addictive disorders. Curr Psychiatry Rep.

[102] Zikos E, Gill KJ, Charney DA (2010). Personality disorders among alcoholic outpatients: Prevalence and course in treatment. Can J Psychiatry.

